# Dietary Supplementation With *Bacillus subtilis* Promotes Growth and Gut Health of Weaned Piglets

**DOI:** 10.3389/fvets.2020.600772

**Published:** 2021-01-15

**Authors:** Zhilong Tian, Xiaodan Wang, Yehui Duan, Yue Zhao, Wenming Zhang, Md. Abul Kalam Azad, Zhanbin Wang, Francois Blachier, Xiangfeng Kong

**Affiliations:** ^1^CAS Key Laboratory of Agro-ecological Processes in Subtropical Region, Hunan Provincial Key Laboratory of Animal Nutritional Physiology and Metabolic Process, National Engineering Laboratory for Pollution Control and Waste Utilization in Livestock and Poultry Production, Institute of Subtropical Agriculture, Chinese Academy of Sciences, Changsha, China; ^2^College of Animal Science and Technology, Henan University of Science and Technology, Luoyang, China; ^3^Evonik (China) Co., Ltd., Beijing, China; ^4^University Paris-Saclay, AgroParisTech, INRAE, UMR PNCA, Paris, France

**Keywords:** *Bacillus subtilis*, growth, gut microbiota, metabolites, intestinal morphology, weaned piglets

## Abstract

This study was conducted to investigate the effects of dietary supplementation with different types of *Bacillus subtilis* (*B. subtilis*) on the growth and gut health of weaned piglets. A total of 160 piglets were randomly assigned into four groups: control group (a basal diet), BS-A group (a basal diet supplemented with *B. subtilis* A at 1 × 10^6^ CFU/g feed), BS-B group (a basal diet supplemented with *B. subtilis* B at 1 × 10^6^ CFU/g feed), and BS-C group (a basal diet supplemented with *B. subtilis* C at 1 × 10^6^ CFU/g feed). All groups had five replicates with eight piglets per replicate. On days 7, 21, and 42 of the trial, blood plasma and intestinal tissues and digesta samples were collected to determine plasma cytokine concentrations, intestinal morphology, gut microbiota community and metabolic activity, and the expression of genes related to gut physiology and metabolism. The results showed that dietary *B. subtilis* supplementation improved (*P* < 0.05) the body weight and average daily gain (in BS-B and BS-C groups) of weaned piglets and decreased (*P* < 0.05) the diarrhea rates (in BS-A, BS-B, and BS-C groups). In the intestinal morphology analysis, *B. subtilis* supplementation improved (*P* < 0.05) the size of villus height and villus height to crypt depth ratio in the ileum of weaned piglets. *Firmicutes, Bacteroidetes*, and *Tenericutes* were the most dominant microflora in piglets' colon whatever the trial group and time of analysis. Dietary BS-C supplementation increased (*P* < 0.05) the relative abundances of *Anaerovibrio* and *Bulleidia* and decreased (*P* < 0.05) the relative abundances of *Clostridium* and *Coprococcus* compared with the control group. In addition, dietary *B. subtilis* supplementation increased (*P* < 0.05) the indicators of intestinal health, including plasma levels of interleukin (IL)-2 and IL-10, as well as the colonic levels of short-chain fatty acids. Furthermore, dietary *B. subtilis* supplementation also up-regulated (*P* < 0.05) the expression of genes involved in metabolic pathways related to intestinal microbiota maturation. In conclusion, these findings suggest that a diet containing BS-B or BS-C can efficiently promote growth performance, decrease diarrhea incidence, and ameliorate several indicators of intestinal health through the modulation of gut microbiota composition and metabolic activity in weaned piglets.

## Introduction

In swine production, weaning is a stressful event that exerts negative effects on microflora balance and mucosal barrier integrity of the digestive tract, eventually leading to gastrointestinal dysfunction, including diarrhea, low feed intake, low weight gain, and poor health of piglets (1). Over the past decades, antibiotics have been widely used as a powerful component to prevent infection and to increase the growth rate (2). However, severe antibiotic resistance problems have been caused by inappropriate or overuse of antibiotics and the widespread use of non-therapeutic antibiotics, leading to a complete ban on the use of antibiotics as growth promoter (3). Therefore, there is an urgent need to develop potential alternatives to growth-promoting antibiotics in order to optimize pig production without negative effects associated with systematic antibiotic use.

Probiotics, as one of the antibiotic alternatives for pig production, are increasingly used as prophylactics for gastrointestinal disorders and as nutritional supplements to promote a satisfactory health status (4). Among several bacterial species used as probiotics, *Bacillus subtilis* (*B. subtilis*) serves as a facultative anaerobe and is widely used as a possible candidate in monogastric feed due to high resistance of its spores to the harsh environment (such as the gastrointestinal tract of animals), and the possibility of long-term storage at ambient temperature (5). More importantly, *B. subtilis*, an intestinal microbe that may grow in the gut, has the ability to consume the oxygen to maintain an anaerobic environment for the prevention or therapy of gastrointestinal disorders (6, 7). Accumulating studies suggest a beneficial role for dietary supplementation with *B. subtilis* (400 g/t diet) on the growth performance of animals through improving intestinal function and health (8). Indeed, dietary supplementation with *B. subtilis* (500 mg/kg diet) showed a beneficial role in maintaining the intestinal barrier function and microflora balance in weaned piglets, and also improved their growth performance (9). However, it is worth to note that since the probiotics' effects are dependent on the combination of selected bacterial genera, their doses, and the feed composition (10), the effects of probiotics on animal growth performance is relatively heterogeneous, depending notably on the overall dietary context (11).

Therefore, we hypothesized that dietary *B. subtilis* supplementation may improve the growth performance and gut health and functions of weaned piglets through modulating the gut microbiota and its metabolic activity. Indeed, several bacterial metabolites have been shown to have an impact on the intestinal epithelium regarding parameters related to energy metabolism, barrier function, and epithelial renewal and homeostasis (12). In this study, we aimed to investigate the effects of three different types of *B. subtilis* on the growth performance, intestinal morphology, and microflora composition of weaned piglets.

## Materials and Methods

### Experimental Design and Dietary Treatments

A total of 160 healthy crossbred piglets (Landrace × Large white, 7.00 ± 0.50 kg body weight) were weaned at 25 days of age and fed a corn and soybean meal-based diet. After 3 days of adaptation, the piglets were randomly assigned into four groups: control group (a basal diet), BS-A group (a basal diet supplemented with *B. subtilis* A at 1 × 10^6^ CFU/g feed), BS-B group (a basal diet supplemented with *B. subtilis* B at 1 × 10^6^ CFU/g feed), and BS-C group (a basal diet supplemented with *B. subtilis* C at 1 × 10^6^ CFU/g feed). All groups had five replicates with eight piglets per replicate. All the probiotics were commercial products provided by Evonik (China), Co., Ltd., Beijing China, and the dose of *B. subtilis* in the piglet's diet was as recommended by the manufacturer. The composition and nutrient levels of the basal diet met the nutritional requirements for nursey piglets established by the National Research Council (13), which are shown in Supplementary Table 1. The experiments lasted for 42 days.

### Determination of Growth Performance and Health Status

All piglets were weighed individually at days 1, 7, 21, and 42 during the trial. The feed consumed by each pen was monitored daily. Average daily gain (ADG), average daily feed intake (ADFI), and feed conversion ratio (FCR; feed consumed/weight gain) were calculated for the periods of 1–7, 8–21, 22–42, and 1–42 days of the trial. The health status of piglets during the trial was assessed by fecal consistency scoring using a four-grade system, where 0 corresponded to firm and dry, 1 to pasty, 2 to thick and fluid, and 3 to watery (14). The fecal score was calculated as the sum of the diarrhea score over the period divided by the number of piglets in the period. The occurrence of diarrhea was defined as maintaining a score of 3 for 1 day. The diarrhea rate (%) was calculated as the sum of the total number of diarrheal piglets over the period divided by the number of piglets in the period multiplied by 100.

### Sample Collection and Preparation

On days 7, 21, and 42 of the trial and 12 h after the last feeding, piglets from each replicate (*n* = 5) close to average body weight (BW) were sampled for plasma analysis and slaughtered by electric shock (120 V, 200 Hz). The intestinal contents from each colon (middle part) were collected and stored at −20°C for analyses of short-chain fatty acids (SCFAs), indole, skatole, bioamines, and the composition of the microbiota. Approximately 2 cm samples of the jejunum (10 cm from the anterior end of the jejunum), ileum (10 cm anterior to the ileocecal valve), and colon tissue (middle part) were collected, washed with cold physiological saline, immediately frozen in liquid nitrogen, and stored at −80°C for further analyses. The jejunum and ileum from all piglets were fixed with 4% paraformaldehyde-PBS for overnight, and then dehydrated and embedded in paraffin blocks. A 5 μm section was cut from each sample for histological analysis.

### Determination of Plasma Cytokines Level

The plasma was collected by centrifuging at 4,000 g for 10 min at 4°C before stored at −20°C. Plasma levels of interleukin (IL)-2, IL-6, IL-10, interferon-alpha (INF-α), and tumor necrosis factor-alpha (TNF-α) were determined using porcine ELISA kits according to the manufacturer's instructions (CUSABIO, Wuhan, China). Plasma cytokine levels were then calculated from the correspondence standard curves.

### Intestinal Histological Examination

The intestinal sections were deparaffinized, hydrated, and stained with hematoxylin and eosin as previously described (15). From each intestinal sample, villus height (VH) and crypt depth (CD) were measured at 10 visual fields, and the villus height to crypt depth ratio (VH/CD) was calculated.

### 16S Sequencing and Bioinformatics Analysis

Microbial genomic DNA was extracted from all samples using a HiPure Stool DNA Kit (Magen, Guangzhou, China) following the manufacturer's instructions. A multiplexed amplicon library covering V3–V4 region of the 16S rDNA gene was PCR-amplified with optimized primer sets for the Illumina HiSeq 2500 sequencing instrument (Illumina, San Diego, CA, USA). Each paired-end read was then spliced using the FLASH (16) software (version 1.2.1) to obtain the original spliced sequence (Raw contigs). Raw tags were mass filtered using Trimmomatic software (version 0.33) to obtain high-quality clean data. All chimeric sequences were removed by Uchime (17) (version 4.2). The chimera-free sequences were processed with a standard QIIME 1.91 pipeline (18) and clustered into operational taxonomic units (OTUs) at a 97% similarity threshold using an “Open-Reference” approach. The raw Illumina pair-end read data for all samples have been deposited in NCBI Sequence Read Archive (SRA) database under accession number: PRJNA597575.

Alpha diversity was analyzed by Chao1, Shannon, and Simpson indexes (19). Beta-diversity was analyzed by principal coordinates analysis (PCoA) based on OTU levels, and the hierarchical clustering tree was constructed based on Unweight Unifrac distances. To decipher the difference in microbiota structure among the four groups, linear discriminant analysis effect size (LEfSe) was performed (20). To probe the microbial metabolism and predict metagenome functional content from the marker genes, PICRUSt was used to explore differences in the KEGG pathway among the four groups (21).

### Analysis of Bacterial Metabolites in Colonic Contents

The colonic contents were collected, homogenized, and centrifuged at 1,000 g for 15 min, as described previously (22). The intestinal SCFAs, including straight-chain fatty acids (acetate, propionate, butyrate, and pentanoate) and branched-chain fatty acids (BCFA; isobutyrate and isopentanoate) were detected by gas chromatography, as described previously (23). The bioamines, including putrescine, tryptamine, tyramine, spermidine, and spermine were measured by high-performance liquid chromatography, as described previously (24). Indole and skatole were analyzed by high-performance liquid chromatography as previously described (22).

### Analysis of mRNA Levels of Genes Related to Intestine Health

Gene expression was measured by real-time polymerase chain reaction (RT-PCR), as previously described (25). Briefly, total RNA was isolated from colonic tissues using TRIzol (Invitrogen, Carlsbad, CA, USA), and fluoresce were monitored by the SYBR Green detection kit (Thermo Fisher Scientific, Waltham, MA, USA) on a 7900 Fast Real-Time PCR System (Applied Biosystems, Foster City, CA, USA). The RT-PCR was conducted with primers of the target genes (Supplementary Table 2) and the reference gene β-actin, and relative gene expression was calculated by the 2^−ΔΔCt^ method (26).

### Statistical Analysis

Intestinal morphology index, colonic metabolite, and the expression of intestinal health-related genes were analyzed with a one-way analysis of variance (ANOVA) using SPSS 19.0 software (SPSS, Inc., Chicago, IL, USA). The data are presented as means ± SE (standard error) and *P* < 0.05 indicates statistical significance. The alpha diversity indices, relative species abundances, and overall composition (at phyla and genera levels) of gut microbial communities were analyzed using the Kruskal-Wallis test. LEfSe was used to identify different taxa microbes among lines using default parameters.

## Results

### Effects of *B. subtilis* on Growth Performance and Diarrhea Rate of Weaned Piglets

The effects of dietary *B. subtilis* supplementation on the growth performance of weaned piglets are presented in Table 1. The piglets in the BS-B and BS-C groups on days 7 and 21 of the trial had higher (*P* < 0.05) BW, as well as the piglets in the BS-C group on day 42, compared with the control group. On the overall experimenatal period (days 1–42), ADG was higher (*P* < 0.05) in the BS-C group than in the control group. In addition, piglets in the BS-B and BS-C groups on days 1–7 of the trial had lower (*P* < 0.05) FCR, as well as the piglets in the BS-C group on days 8–21, compared with the control group. Moreover, dietary *B. subtilis* supplementation (with the BS-A, BS-B, or BS-C types) decreased (*P* < 0.05) the diarrhea rate on the overall experimental period (days 1–42), except for the BS-C group on days 1–7 of the trial, compared with the control group.

**Table 1:** Effect of dietary supplementation with different types of *B. subtilis* on growth performance and diarrhea rate of weaned piglets.

**Items**	**Control group**	**BS-A group**	**BS-B group**	**BS-C group**
**Body weight (kg)**				
Day 1	7.02 ± 0.07	7.08 ± 0.04	7.09 ± 0.04	7.07 ± 0.05
Day 7	8.47 ± 0.08^c^	8.72 ± 0.05^b^	9.06 ± 0.04^a^	8.95 ± 0.08^a^
Day 21	13.42 ± 0.31^c^	13.73 ± 0.25^b^^c^	14.41 ± 0.33^a^^b^	14.79 ± 0.11^a^
Day 42	23.28 ± 0.15^b^	24.09 ± 0.23^a^^b^	24.75 ± 0.42^a^^b^	25.31 ± 0.07^a^
**Average daily gain (kg/d)**				
Day 1–7	0.21 ± 0.01^b^	0.23 ± 0.01^b^	0.28 ± 0.00^a^	0.27 ± 0.02^a^
Day 8–21	0.35 ± 0.02^b^	0.36 ± 0.02^b^	0.38 ± 0.02^a^^b^	0.42 ± 0.01^a^
Day 22–42	0.47 ± 0.01	0.49 ± 0.01	0.49 ± 0.02	0.50 ± 0.01
Day 1–42	0.39 ± 0.00^c^	0.40 ± 0.01^b^^c^	0.42 ± 0.01^a^^b^	0.43 ± 0.00^a^
**Average daily feed intake (g/d)**				
Day 1–7	392.51 ± 5.34	395.54 ± 11.73	422.19 ± 14.93	432.87 ± 26.48
Day 8–21	585.47 ± 10.94	594.68 ± 22.57	563.92 ± 45.67	596.76 ± 15.72
Day 22–42	859.50 ± 55.65	941.08 ± 41.91	903.88 ± 49.56	915.31 ± 49.52
Day 1–42	690.32 ± 31.58	734.69 ± 28.48	710.28 ± 36.84	728.72 ± 33.20
**Feed conversion ratio**				
Day 1–7	1.91 ± 0.11^a^	1.70 ± 0.05^a^^b^	1.50 ± 0.05^b^	1.62 ± 0.06^b^
Day 8–21	1.69 ± 0.11^a^	1.66 ± 0.03^a^	1.47 ± 0.05^a^^b^	1.44 ± 0.07^b^
Day 22–42	1.83 ± 0.10	1.91 ± 0.10	1.85 ± 0.12	1.83 ± 0.09
Day 1–42	1.78 ± 0.08	1.81 ± 0.06	1.69 ± 0.07	1.68 ± 0.07
**Diarrhea rate (%)**				
Day 1–7	5.71 ± 0.63^a^	4.14 ± 0.31^b^	4.43 ± 0.24^b^	4.57 ± 0.31^a^^b^
Day 8–21	3.26 ± 0.23^a^	2.01 ± 0.13^b^	2.12 ± 0.07^b^	2.23 ± 0.20^b^
Day 22–42	1.48 ± 0.13^a^	0.58 ± 0.06^b^	0.38 ± 0.09^b^	0.35 ± 0.11^b^
Day 1–42	3.48 ± 0.22^a^	2.24 ± 0.11^b^	2.31 ± 0.08^b^	2.39 ± 0.17^b^

*Values are expressed as means ± SE, n = 5*.

a,b,c *Mean values within a same row with different superscript letters were significantly different (P < 0.05). Control group, a basal diet; BS-A group, a basal diet supplemented with B. subtilis A at 1 × 10^6^ CFU/g feed; BS-B group, a basal diet supplemented with B. subtilis B at 1 × 10^6^ CFU/g feed; and BS-C group, a basal diet supplemented with B. subtilis C at 1 × 10^6^ CFU/g feed*.

### Effects of *B. subtilis* on Plasma Cytokine Levels of Weaned Piglets

The effects of dietary *B. subtilis* supplementation on plasma cytokine levels are presented in Table 2. The dietary supplementation resulted in some changes in the cytokine levels that were on some occasion transient. Compared with the control group, dietary BS-B supplementation increased (*P* < 0.05) plasma levels of IL-2 and TNF-α on day 7 of the trial, as well as the IL-10 level on day 21 of the trial. Dietary supplementation with three kinds of *B. subtilis* increased (*P* < 0.05) the IL-2 level and decreased (*P* < 0.05) the TNF-α level on day 21 of the trial. The BS-C supplementation increased (*P* < 0.05) the plasma levels of IL-6, IL-10, and INF-α on day 21 of the trial. On day 42 of the trial, the BS-A and BS-B groups showed an increased (*P* < 0.05) plasma IL-2 level compared with the control group.

**Table 2:** Effect of dietary supplementation with different types of *B. subtilis* on plasma cytokine levels of weaned piglets (pg/mL).

**Items**	**Control group**	**BS-A group**	**BS-B group**	**BS-C group**
**Day 7**				
IL-2	89.12 ± 18.55^b^	145.41 ± 29.51^a^^b^	177.63 ± 30.38^a^	133.70 ± 5.81^a^^b^
IL-6	205.54 ± 46.28	198.21 ± 24.06	219.48 ± 20.48	273.91 ± 22.57
IL-10	48.28 ± 13.59	63.67 ± 19.81	74.61 ± 22.40	49.09 ± 7.83
INF-α	63.78 ± 7.71	91.16 ± 22.72	62.84 ± 6.06	61.58 ± 3.55
TNF-α	27.05 ± 1.99^b^	49.47 ± 0.61^a^^b^	65.94 ± 9.36^a^	45.63 ± 15.82^a^^b^
**Day 21**				
IL-2	61.37 ± 7.09^c^	138.42 ± 21.14^b^	174.30 ± 2.87^a^^b^	208.07 ± 28.49^a^
IL-6	145.08 ± 11.72^b^	293.15 ± 54.20^a^^b^	324.25 ± 63.15^a^^b^	414.42 ± 92.45^a^
IL-10	26.70 ± 1.13^b^	40.83 ± 7.53^a^^b^	55.75 ± 6.47^a^	58.31 ± 12.22^a^
INF-α	55.52 ± 6.94^a^^b^	40.47 ± 5.67^b^	64.91 ± 9.61^a^^b^	86.48 ± 19.84^a^
TNF-α	142.00 ± 33.07^a^	30.82 ± 1.88^b^	41.08 ± 12.82^b^	66.34 ± 9.86^b^
**Day 42**				
IL-2	90.16 ± 27.11^b^	153.35 ± 17.10^a^	175.58 ± 3.90^a^	146.34 ± 18.41^a^^b^
IL-6	188.31 ± 51.86	311.61 ± 55.40	329.76 ± 50.13	250.95 ± 38.84
IL-10	37.65 ± 15.62	29.98 ± 8.33	32.39 ± 12.01	36.61 ± 4.21
INF-α	43.30 ± 14.45	39.99 ± 6.97	30.75 ± 5.43	48.06 ± 6.56
TNF-α	91.50 ± 19.95	67.67 ± 15.21	45.44 ± 14.79	53.25 ± 11.49

*Values are presented as means ± SE, n = 5*.

a,b,c *Mean values within the same row with different superscript letters were significantly different (P < 0.05). IL, interleukin; IFN-α, interferon-alpha; TNF-α, tumor necrosis factor-alpha. Control group, a basal diet; BS-A group, a basal diet supplemented with B. subtilis A at 1 × 10^6^ CFU/g feed; BS-B group, a basal diet supplemented with B. subtilis B at 1 × 10^6^ CFU/g feed; and BS-C group, a basal diet supplemented with B. subtilis C at 1 × 10^6^ CFU/g feed*.

### Effects of *B. subtilis* on Intestinal Morphology of Weaned Piglets

The effects of dietary *B. subtilis* supplementation on intestinal morphology are presented in Table 3. On day 7 of the trial, the BS-C supplementation increased (*P* < 0.05) VH, as well as the BS-A, BS-B, and BS-C supplementation on VH/CD ratio, while the BS-A and BS-B supplementation decreased (*P* < 0.05) CD in the jejunum, compared with the control group. On days 21 and 42 of the trial, the BS-B supplementation increased (*P* < 0.05) VH in the jejunum compared with the control group. In the ileum, dietary supplementation with BS-A, BS-B, or BS-C increased (*P* < 0.05) VH throughout the trial and VH/CD ratio on days 7 and 42, compared with the control group. However, on day 21 of the trial, the BS-C supplementation increased (*P* < 0.05) VH/CD ratio and the BS-B supplementation decreased (*P* < 0.05) CD compared with the control group. Interestingly, an increase in the jejunum and ileum CD was observed in the BS-B group relative to the control group on day 21 of the trial (*P* < 0.05).

**Table 3:** Effect of dietary supplementation with different types of *B. subtilis* on intestinal morphology of weaned piglets.

**Items**		**Control group**	**BS-A group**	**BS-B group**	**BS-C group**
**Day 7**					
Jejunum	VH (μm)	334.42 ± 6.77^b^	310.29 ± 29.05^b^	314.59 ± 10.36^b^	393.23 ± 12.92^a^
	CD (μm)	291.98 ± 31.40^a^	190.87 ± 15.49^b^	199.73 ± 18.35^b^	234.23 ± 8.32^a^^b^
	VH/CD	1.20 ± 0.13^b^	1.62 ± 0.05^a^	1.74 ± 0.09^a^	1.69 ± 0.07^a^
Ileum	VH (μm)	304.68 ± 19.15^b^	377.07 ± 9.20^a^	397.52 ± 18.78^a^	395.08 ± 19.61^a^
	CD (μm)	171.76 ± 4.10	163.24 ± 4.78	160.12 ± 12.49	182.20 ± 8.31
	VH/CD	1.78 ± 0.14^b^	2.32 ± 0.09^a^	2.51 ± 0.11^a^	2.19 ± 0.13^a^
**Day 21**					
Jejunum	VH (μm)	357.82 ± 15.15^b^	429.22 ± 32.41^a^^b^	478.66 ± 30.76^a^	430.91 ± 6.76^a^^b^
	CD (μm)	260.30 ± 19.98^b^	270.18 ± 18.49^b^	335.63 ± 26.46^a^	298.17 ± 16.36^a^^b^
	VH/CD	1.41 ± 0.13	1.59 ± 0.09	1.44 ± 0.06	1.53 ± 0.10
Ileum	VH (μm)	362.35 ± 7.42^c^	401.45 ± 19.11^b^	480.95 ± 13.14^a^	442.94 ± 8.70^a^
	CD (μm)	174.85 ± 3.86^b^	179.86 ± 10.52^b^	233.19 ± 8.26^a^	171.97 ± 9.67^b^
	VH/CD	2.08 ± 0.06^b^	2.25 ± 0.13^b^	2.07 ± 0.08^b^	2.61 ± 0.14^a^
**Day 42**					
Jejunum	VH (μm)	408.60 ± 4.41^b^	461.22 ± 44.99^a^^b^	529.98 ± 25.19^a^	476.11 ± 9.85^a^^b^
	CD (μm)	296.57 ± 16.46	341.39 ± 8.68	339.65 ± 27.88	296.06 ± 21.71
	VH/CD	1.39 ± 0.06	1.48 ± 0.10	1.58 ± 0.07	1.65 ± 0.13
Ileum	VH (μm)	338.67 ± 7.07^b^	474.40 ± 21.70^a^	446.03 ± 31.09^a^	461.42 ± 29.41^a^
	CD (μm)	206.36 ± 11.20^a^^b^	186.39 ± 18.78^a^^b^	174.86 ± 12.20^b^	221.66 ± 13.17^a^
	VH/CD	1.54 ± 0.04^c^	2.62 ± 0.22^a^	2.56 ± 0.12^a^	2.08 ± 0.04^b^

*Data are presented as means ± SE, n = 5*.

a,b,c *Mean values within the same row with different superscript letters were significantly different (P < 0.05). VH, villus height; CD, crypt depth; VH/CD, villus height to crypt depth ratio. Control group, a basal diet; BS-A group, a basal diet supplemented with B. subtilis A at 1 × 10^6^ CFU/g feed; BS-B group, a basal diet supplemented with B. subtilis B at 1 × 10^6^ CFU/g feed; and BS-C group, a basal diet supplemented with B. subtilis C at 1 × 10^6^ CFU/g feed*.

### Effects of *B. subtilis* on Microbiota Diversity of Weaned Piglets

**Figure 1:**
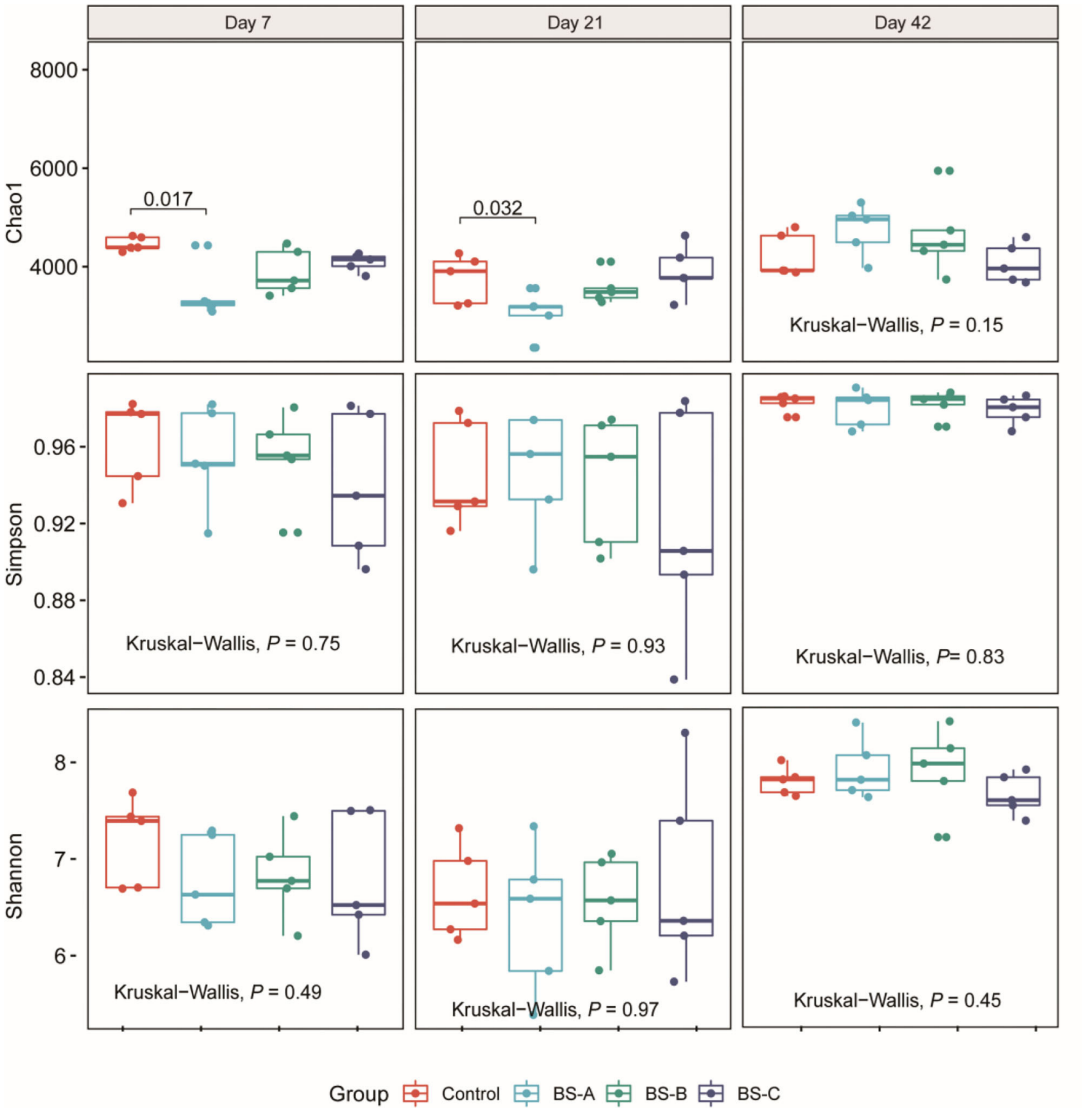
Alpha diversity of the colonic bacterial community of weaned piglets fed with different types of *B. subtilis* (*n* = 5). Control group, a basal diet; BS-A group, a basal diet supplemented with *B. subtilis* A at 1 × 10^6^ CFU/g feed; BS-B group, a basal diet supplemented with *B. subtilis* B at 1 × 10^6^ CFU/g feed; and BS-C group, a basal diet supplemented with *B. subtilis* C at 1 × 10^6^ CFU/g feed.

**Figure 2:**
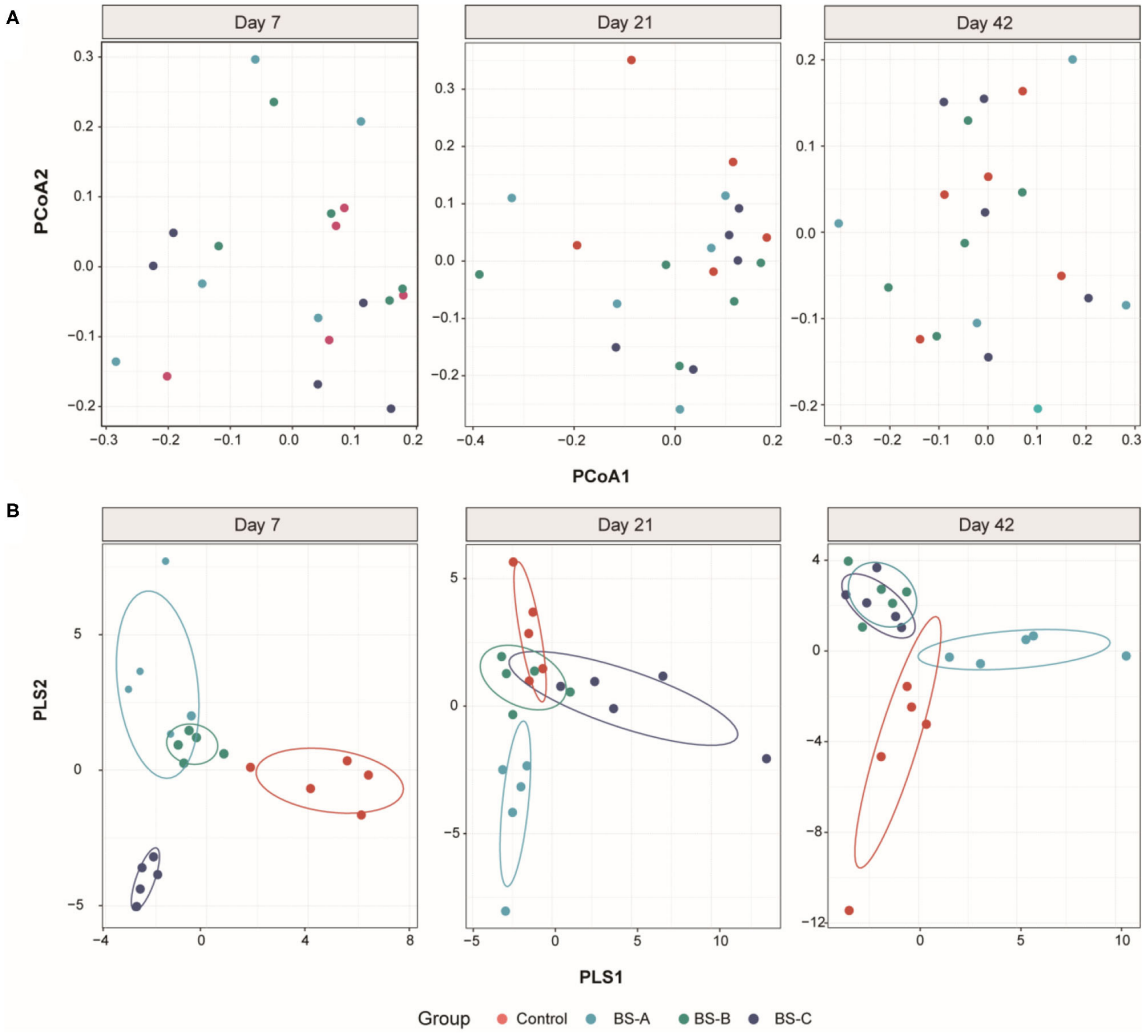
Beta diversity of the colonic bacterial community of weaned piglets fed with different types of *B. subtilis* (*n* = 5). Principal coordinate analysis (PCoA) on unweighted uniFrac distances among the four groups is shown along with the first two principal coordinates (PC) axes **(A)**. Partial least square discriminant analysis (PLS-DA) score plots among the four groups **(B)**. Control group, a basal diet; BS-A group, a basal diet supplemented with *B. subtilis* A at 1 × 10^6^ CFU/g feed; BS-B group, a basal diet supplemented with *B. subtilis* B at 1 × 10^6^ CFU/g feed; and BS-C group, a basal diet supplemented with *B. subtilis* C at 1 × 10^6^ CFU/g feed.

The effects of dietary *B. subtilis* supplementation on colonic microbial communities are presented in Figure 1. There were no significant differences in the Simpson or Shannon indexes throughout the trial (*P* > 0.05). However, the BS-A group had a lower (*P* < 0.05) Chao1 index than in the control group on days 7 and 21 of the trial. The PCoA analysis at the OTU level showed that there was no separation between the four groups (Figure 2A). Further investigation by using partial least square discriminant analysis (PLS-DA) as a supervised analysis showed that the microbial community structure was clearly separated and clustered into four groups (Figure 2B) during the overall experimental period.

### Effects of *B. subtilis* on Microbial Communities of Weaned Piglets

**Figure 3:**
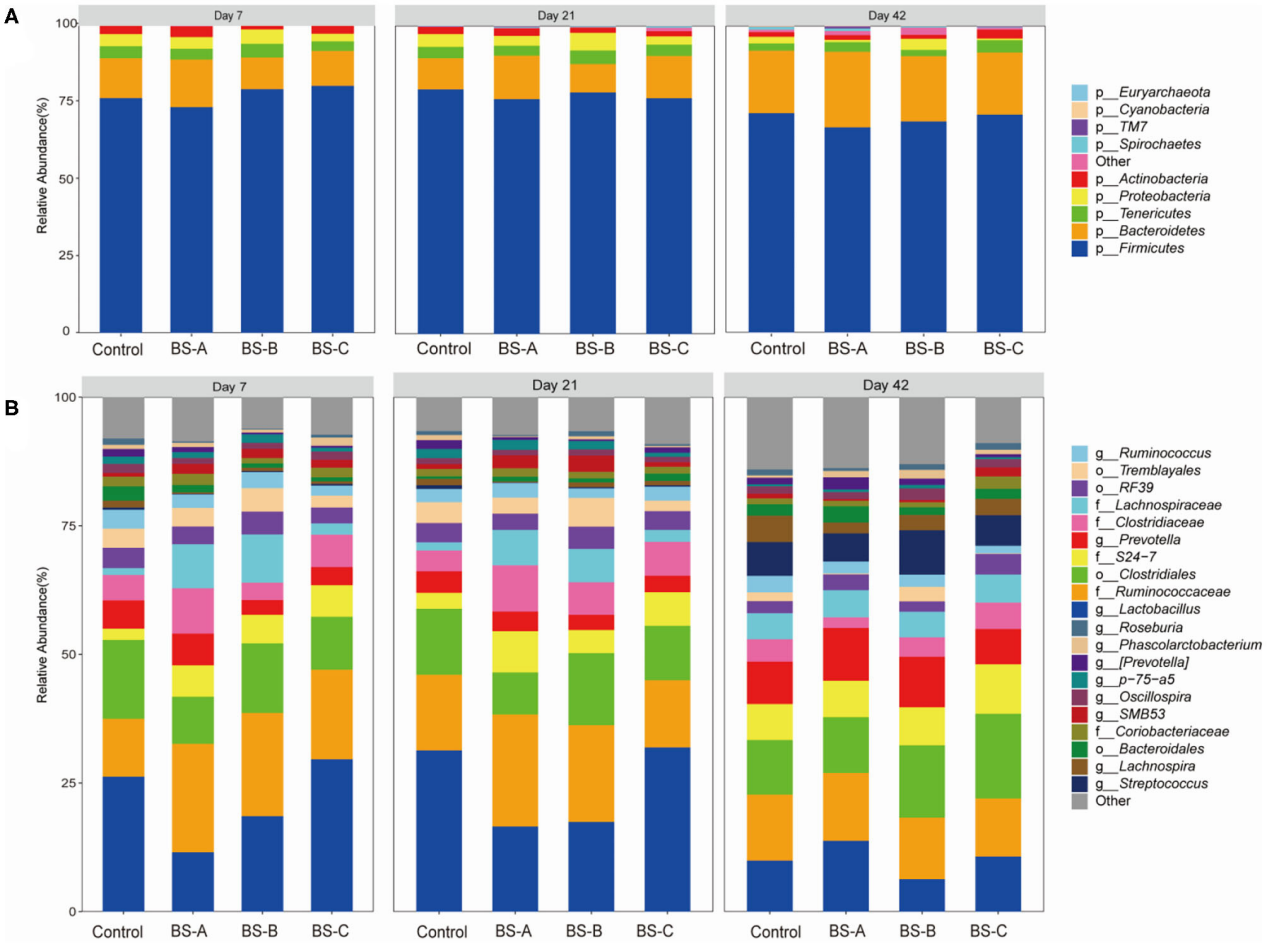
Colonic microbiota composition of weaned piglets fed with different types of *B. subtilis* (*n* = 5). Microbial community bar plot at the phylum level **(A)** and genus level **(B)**. Control group, a basal diet; BS-A group, a basal diet supplemented with *B. subtilis* A at 1 × 10^6^ CFU/g feed; BS-B group, a basal diet supplemented with *B. subtilis* B at 1 × 10^6^ CFU/g feed; and BS-C group, a basal diet supplemented with *B. subtilis* C at 1 × 10^6^ CFU/g feed.

Taxonomic differences in the microbial composition of weaned piglet's colonic contents showed that *Firmicutes* (67–80%), *Bacteroidetes* (10–25%), *Tenericutes* (2–5%), and *Proteobacteria* (1–6%) were the most abundant bacterial phyla for the overall experimental period (> 95% of OTUs) (Figure 3A). On days 21 and 42 of the trial, dietary BS-A, BS-B, or BS-C supplementation decreased (*P* < 0.05) the relative abundance of *Firmicutes* whereas increased (*P* < 0.05) the relative abundance of *Bacteroidetes* on day 42 of the trial compared with the control group. The top four abundant genera in the control group were *g_Lactobacillus* (>26%), f_*Ruminococcaceae* (>11%), o_*Clostridiales* (>13%), and *Prevotella* (>4%) on days 7 and 21 of the trial (Figure 3B). The BS-A and BS-B supplementation increased the relative abundance of *Ruminococcaceae* but decreased the relative abundance of *Lactobacillus* compared with the control group (*P* < 0.05). On day 42 of the trial, the top four abundant genera were *f_Ruminococcaceae* (13%), *o_Clostridiales* (11%), *Lactobacillus* (10%), and *Prevotella* (9%) in the control group. The BS-A supplementation increased (*P* < 0.05) the relative abundances of *o_Clostridiales, f_Ruminococcaceae, Lactobacillus*, and *Prevotella* compared with the control group.

**Figure 4:**
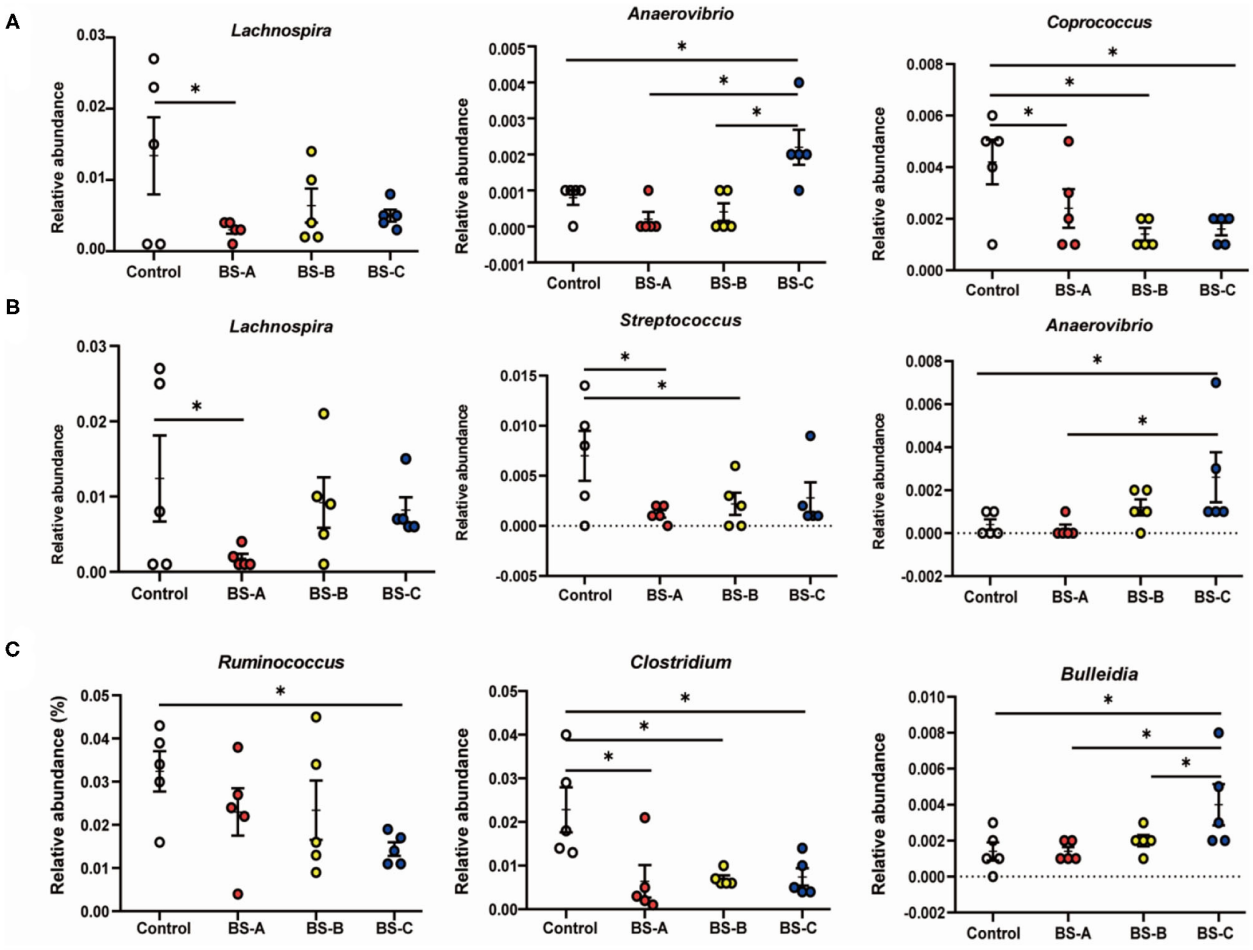
Comparison of the colonic microbial community of weaned piglets fed with different types of *B. subtilis* on days 7 **(A)**, 21 **(B)**, and 42 **(C)** of the trial, respectively. Values are expressed as means ± SE, *n* = 5. *P < 0.05. Control group, a basal diet; BS-A group, a basal diet supplemented with *B. subtilis* A at 1 × 10^6^ CFU/g feed; BS-B group, a basal diet supplemented with *B. subtilis* B at 1 × 10^6^ CFU/g feed; and BS-C group, a basal diet supplemented with *B. subtilis* C at 1 × 10^6^ CFU/g feed.

Taxonomic differences of the colonic microbiota at the genus level are shown in Figure 4. On days 7 and 21 of the trial, the BS-A supplementation decreased (*P* < 0.05) the relative abundance of *Lachnospira* compared with the control group. Meanwhile, the relative abundance of *Anaerovibrio* was higher (*P* < 0.05) in the BS-C group than in the BS-A and control groups. On day 7 of the trial, the relative abundance of *Coprococcus* was lower (*P* < 0.05) in the three *B*. *subtilis* supplemented groups than in the control group (Figure 4A). On day 21 of the trial, the relative abundance of *Streptococcus* was lower (*P* < 0.05) in the BS-A and BS-B groups than in the control group (Figure 4B). On day 42 of the trial, *Ruminococcus* showed a lower abundance in the BS-C group than in the control group (*P* < 0.05) (Figure 4C). Furthermore, the relative abundance of *Clostridium* was lower (*P* < 0.05) in the three *B*. *subtilis* supplemented groups than in the control group, while *Bulleidia* showed a higher (*P* < 0.05) abundance in the BS-C group than in the other three groups (Figure 4C).

### Effects of *B. subtilis* on Microbial Function of Weaned Piglets

**Figure 5:**
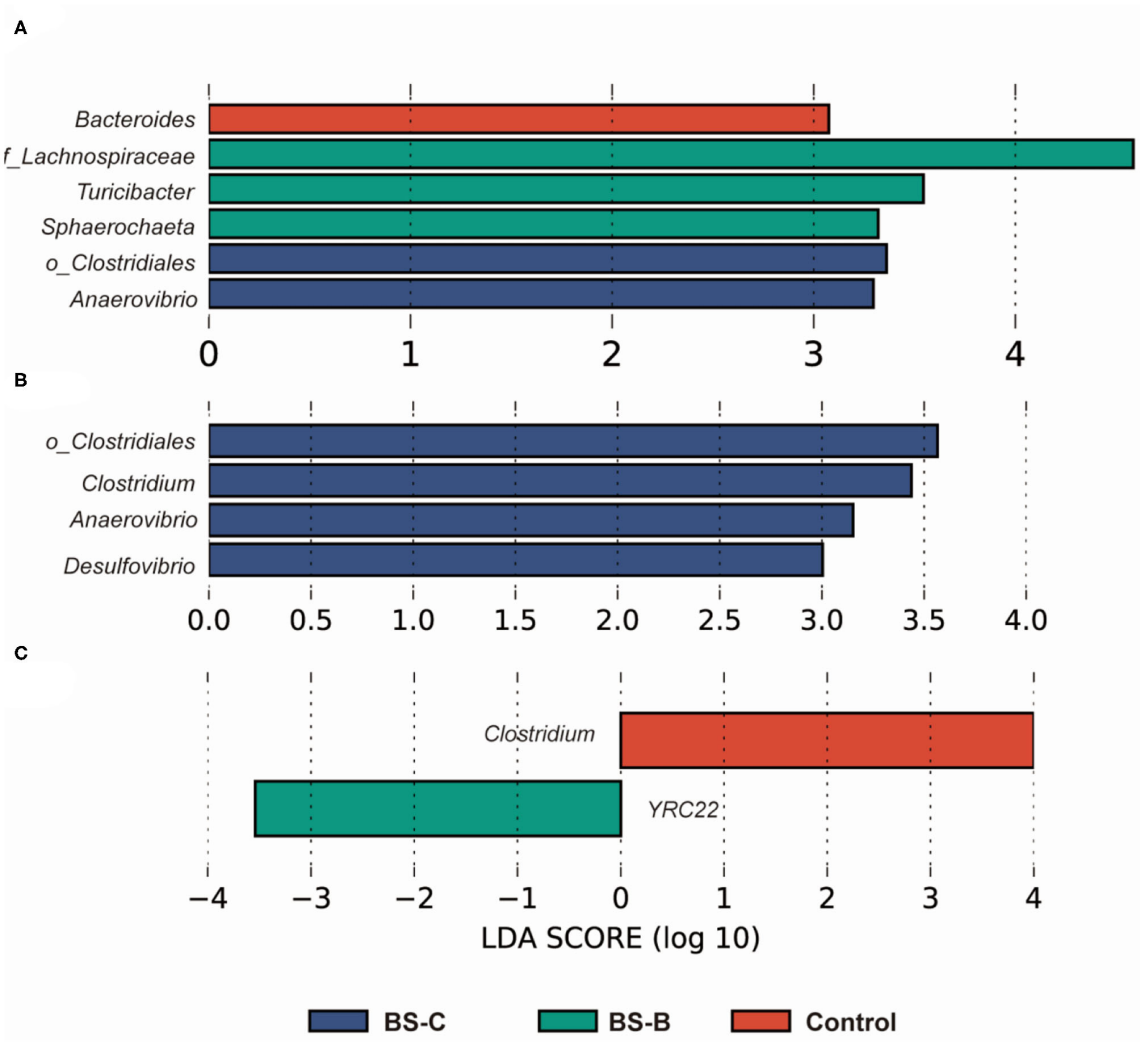
LEfSe analysis of the colonic microbial community of weaned piglets fed with different types of *B. subtilis* on days 7 **(A)**, 21 **(B)**, and 42 **(C)** of the trial, respectively (*n* = 5). Control group, a basal diet; BS-A group, a basal diet supplemented with *B. subtilis* A at 1 × 10^6^ CFU/g feed; BS-B group, a basal diet supplemented with *B. subtilis* B at 1 × 10^6^ CFU/g feed; and BS-C group, a basal diet supplemented with *B. subtilis* C at 1 × 10^6^ CFU/g feed.

The LEfSe analysis was carried out to understand the impacts of *B. subtilis* on the intestinal microbiota of piglets (Figure 5). The data showed significant differences among the four groups in the colonic microbiota for the entire trial. On day 7 of the trial, *Lachnospiraceae, Turicibacter*, and *Sphaerochaeta* were enriched in the BS-B group and *Clostridiales* and *Anaerovibrio* were enriched in the BS-C group (Figure 5A). *Clostridiales, Anaerovibrio*, and *Desulfovibrio* were the most abundant in the BS-C group on day 21 of the trial (Figure 5B). Morevoer, *Clostridium* was enriched in the control group and *YRC*-22 was enriched in the BS-B group on day 42 of the trial (Figure 5C).

**Figure 6:**
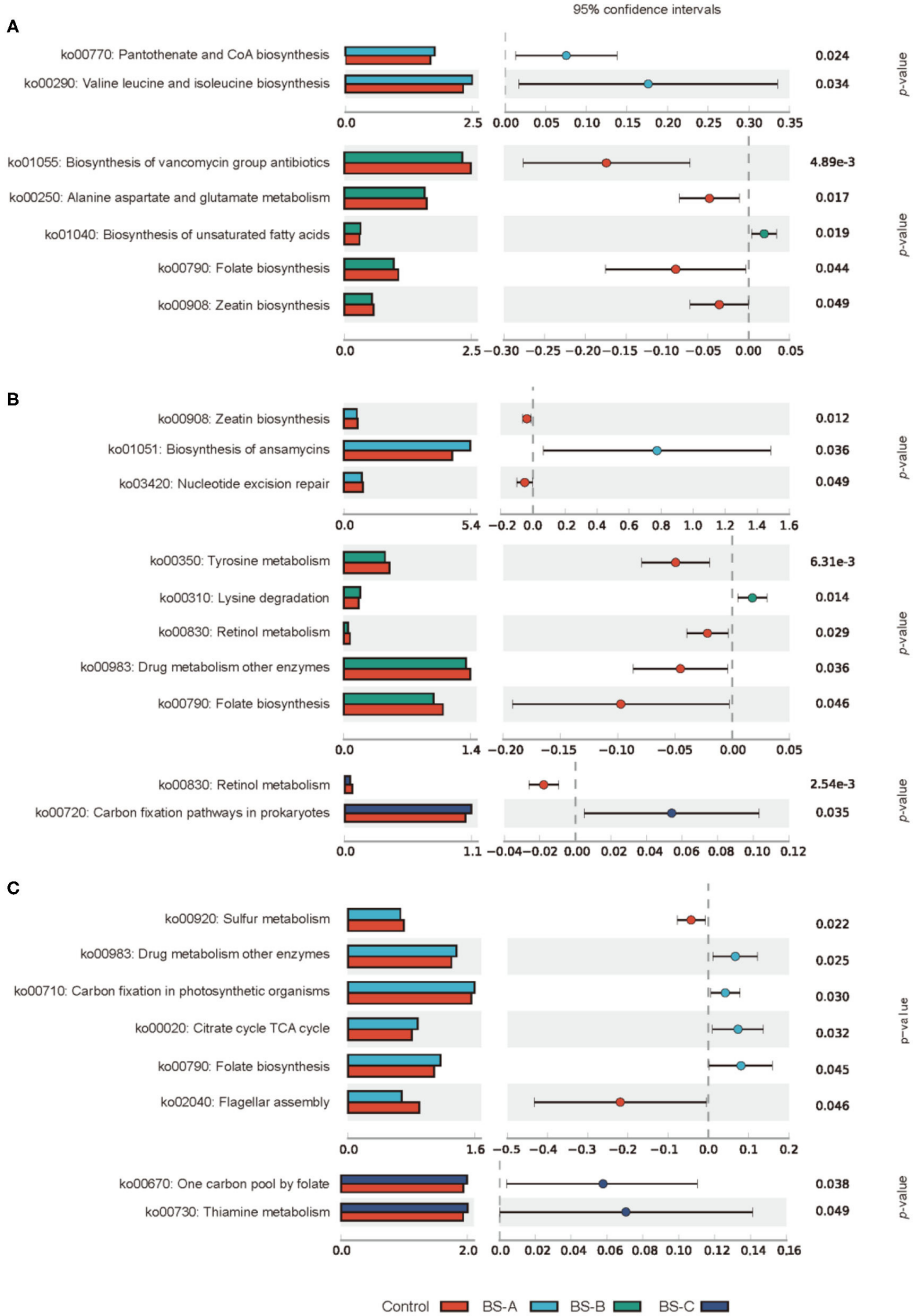
PICRUSt and KEGG analysis of the colonic microbial community of weaned piglets fed with different types of *B. subtilis* on days 7 **(A)**, 21 **(B)**, and 42 **(C)** of the trial, respectively (*n* = 5). Control group, a basal diet; BS-A group, a basal diet supplemented with *B. subtilis* A at 1 × 10^6^ CFU/g feed; BS-B group, a basal diet supplemented with *B. subtilis* B at 1 × 10^6^ CFU/g feed; and BS-C group, a basal diet supplemented with *B. subtilis* C at 1 × 10^6^ CFU/g feed.

The PICRUSt algorithm was used to determine the functional differences by plotting different pathways against the KEGG database (Figure 6). On day 7 of the trial, the intestinal microbiota-enriched pathways in the three *B. subtilis* supplemented groups included metabolic steps involved in the biosynthesis of pantothenate and CoA; valine, leucine, and isoleucine biosynthesis; and unsaturated fatty acids (Figure 6A). On day 21 of the trial, the intestinal microbiota-enriched pathways in the three *B. subtilis* supplemented groups, included metabolic steps involved in biosynthesis of ansamycins, lysine degradation, and carbon fixation in prokaryotes (Figure 6B). On day 42 of the trial, the pathways involved in the three *B. subtilis* supplemented groups, are notably related to drug metabolism, carbon fixation in photosynthetic organisms, citrate/TCA cycle, folate biosynthesis, folate-dependent one-carbon metabolism, and thiamine metabolism (Figure 6C).

### Effects of *B. subtilis* on Gut Metabolite Concentrations in the Colon of Weaned Piglets

The effects of dietary *B. subtilis* supplementation on colonic SCFA concentrations are presented in Table 4. The acetate concentration was decreased (*P* < 0.05) in the BS-B group compared with the BS-A group on day 7 of the trial, as well as the propionate concentration compared with the BS-C group. On day 21 of the trial, the isobutyrate and isovalerate concentrations were increased (*P* < 0.05) in the BS-C group relative to the control group, as well as the butyrate concentration in the BS-C and control groups compared with the BS-B group. On day 42 of the trial, the acetate concentration was increased (*P* < 0.05) in the BS-B and BS-C groups, while the concentrations of isovalerate and valerate were decreased (*P* < 0.05) in the BS-B group, when compared with the control group.

**Table 4:** Effect of dietary supplementation with different types of *B. subtilis* on short-chain fatty acid concentration of colonic contents of weaned piglets (μg/g).

**Items**	**Control group**	**BS-A group**	**BS-B group**	**BS-C group**
**Day 7**				
Acetate	3.12 ± 0.17^a^^b^	3.31 ± 0.25^a^	2.60 ± 0.21^b^	2.78 ± 0.08^a^^b^
Propionate	1.12 ± 0.03^a^^b^	1.41 ± 0.14^a^^b^	1.09 ± 0.10^b^	1.74 ± 0.36^a^
Butyrate	0.80 ± 0.06	0.80 ± 0.08	0.69 ± 0.04	1.01 ± 0.21
Isobutyrate	0.07 ± 0.01	0.07 ± 0.01	0.08 ± 0.01	0.09 ± 0.02
Valerate	0.18 ± 0.05	0.15 ± 0.02	0.10 ± 0.01	0.16 ± 0.04
Isovalerate	0.11 ± 0.02	0.12 ± 0.02	0.14 ± 0.02	0.15 ± 0.03
**Day 21**				
Acetate	3.06 ± 0.05	3.25 ± 0.27	2.64 ± 0.22	3.06 ± 0.16
Propionate	1.61 ± 0.05	1.67 ± 0.12	1.55 ± 0.03	1.73 ± 0.19
Butyrate	1.13 ± 0.09^a^^b^	0.98 ± 0.10^b^^c^	0.86 ± 0.06^c^	1.35 ± 0.03^a^
Isobutyrate	0.10 ± 0.01^b^	0.13 ± 0.01^a^^b^	0.11 ± 0.01^a^^b^	0.14 ± 0.01^a^
Valerate	0.21 ± 0.03	0.24 ± 0.04	0.24 ± 0.05	0.28 ± 0.05
Isovalerate	0.17 ± 0.01^b^	0.24 ± 0.03^a^^b^	0.21 ± 0.03^a^^b^	0.25 ± 0.03^a^
**Day 42**				
Acetate	2.23 ± 0.20^b^	2.61 ± 0.13^a^^b^	3.00 ± 0.12^a^	2.79 ± 0.18^a^
Propionate	1.09 ± 0.16	0.99 ± 0.06	1.27 ± 0.06	1.17 ± 0.13
Butyrate	0.87 ± 0.12	0.73 ± 0.11	0.92 ± 0.01	0.68 ± 0.03
Isobutyrate	0.13 ± 0.03	0.09 ± 0.00	0.08 ± 0.00	0.08 ± 0.00
Valerate	0.22 ± 0.07^a^	0.10 ± 0.00^b^	0.11 ± 0.00^b^	0.13 ± 0.01^a^^b^
Isovalerate	0.24 ± 0.07^a^	0.14 ± 0.01^a^^b^	0.12 ± 0.00^b^	0.14 ± 0.01^a^^b^

*Values are expressed as means ± SE, n = 5*.

a,b,c *Mean values within the same row with different superscript letters were significantly different (P < 0.05). Control group, a basal diet; BS-A group, a basal diet supplemented with B. subtilis A at 1 × 10^6^ CFU/g feed; BS-B group, a basal diet supplemented with B. subtilis B at 1 × 10^6^ CFU/g feed; and BS-C group, a basal diet supplemented with B. subtilis C at 1 × 10^6^ CFU/g feed*.

As shown in Supplementary Table 3, the bioamine concentrations of colonic contents from the four groups were not significantly different on days 7 and 42 of the trial (*P* > 0.05). The tryptamine concentration was decreased (*P* < 0.05) in the BS-C group compared with the control group on day 21 of the trial, as well as the tyramine concentration compared with the BS-A group. Compared with the control group, the skatole concentration was decreased (*P* < 0.05) in the three *B*. *subtilis* supplemented groups on day 42 of the trial, as well as the skatole concentration in the BS-A group on day 21 of the trial. Moreover, the indole concentration was increased (*P* < 0.05) in the BS-A group compared with the control group on day 42 of the trial.

### Effect of *B. subtilis* on mRNA Levels of Intestinal Health-related Genes of Weaned Piglets

The effects of dietary *B. subtilis* supplementation on the intestinal health-related gene mRNA levels are presented in Table 5. Compared with the control group, the mRNA level of *E-cadherin* was up-regulated (*P* < 0.05) in the three *B. subtilis* supplemented groups during the whole trial period. On day 7 of the trial, the levels of IL-6 and *occludin* were up-regulated (*P* < 0.05) in the three *B. subtilis* supplemented groups, as well as zonula occuldens (*ZO*)-1 and IL-10 in the BS-A and BS-B groups, when compared with the control group. Furthermore, compared with the control group, the mRNA levels of IL-1β and toll-like receptor (TLR)-4 were up-regulated (*P* < 0.05) in the BS-A and BS-B groups, as well as IL-2 and IFN-α in the BS-A and BS-C groups. On day 21 of the trial, compared with the control group, the level of IFN-α in the BS-A and BS-B groups and the level of IL-6 in the BS-B and BS-C groups was up-regulated (*P* < 0.05), as well as the level of IL-2 in the BS-A group and the level of TNF-α in the BS-B group. On day 42 of the trial, the mRNA levels of IL-β, IL-2, IL-10, and TLR-4 were up-regulated (*P* < 0.05) in the three *B. subtilis* supplemented groups compared with the control group. Moreover, the levels of IL-6 and TNF-α were up-regulated (*P* < 0.05) in the BS-B group, as well as the level of *ZO-*1 in the BS-B and BS-C groups and the level of *occludin* in the BS-C group, when compared with the control group.

**Table 5:** Effect of dietary supplementation with different types of *B. subtilis* on health-related genes of weaned piglets.

**Items**	**Control group**	**BS-A group**	**BS-B group**	**BS-C group**
**Day 7**				
IL-1β	1.00 ± 0.06^b^	1.57 ± 0.31^a^	1.88 ± 0.06^a^	1.38 ± 0.12^a^^b^
IL-2	1.00 ± 0.12^b^	1.39 ± 0.04^a^	1.18 ± 0.10^a^^b^	1.46 ± 0.03^a^
IL-6	1.00 ± 0.10^c^	1.47 ± 0.12^b^	1.75 ± 0.08^a^	1.83 ± 0.05^a^
IL-10	1.00 ± 0.17^b^	1.88 ± 0.44^a^^b^	1.98 ± 0.18^a^	1.54 ± 0.24^a^^b^
IFN-α	1.02 ± 0.12^b^	1.31 ± 0.04^a^	1.10 ± 0.09^a^^b^	1.37 ± 0.03^a^
TNF-α	1.00 ± 0.13	1.90 ± 0.47	1.68 ± 0.10	1.74 ± 0.41
TLR-4	1.00 ± 0.06^b^	1.48 ± 0.06^a^	1.46 ± 0.16^a^	1.29 ± 0.09^a^^b^
*E-cadherin*	1.00 ± 0.08^b^	1.23 ± 0.01^a^	1.32 ± 0.06^a^	1.24 ± 0.01^a^
*Occludin*	1.00 ± 0.08^b^	1.66 ± 0.26^a^	2.00 ± 0.14^a^	1.87 ± 0.21^a^
*ZO-*1	1.00 ± 0.08^b^	1.90 ± 0.29^a^	1.49 ± 0.17^a^^b^	1.16 ± 0.08^b^
**Day 21**				
IL-1β	1.00 ± 0.25	1.37 ± 0.27	1.32 ± 0.06	1.33 ± 0.28
IL-2	0.69 ± 0.06^b^	1.69 ± 0.29^a^	1.35 ± 0.31^a^^b^	1.17 ± 0.05^a^^b^
IL-6	1.00 ± 0.07^b^	1.38 ± 0.19^b^	1.50 ± 0.12^a^	1.63 ± 0.03^a^
IL-10	1.00 ± 0.24	1.30 ± 0.15	1.15 ± 0.14	1.22 ± 0.11
IFN-α	1.00 ± 0.17^b^	1.64 ± 0.08^a^	1.65 ± 0.11^a^	0.93 ± 0.07^b^
TNF-α	1.00 ± 0.04^b^	1.93 ± 0.35^a^^b^	2.62 ± 0.50^a^	1.59 ± 0.26^a^^b^
TLR-4	1.00 ± 0.15	1.57 ± 0.30	1.28 ± 0.18	1.03 ± 0.07
*E-cadherin*	0.90 ± 0.02^b^	1.37 ± 0.18^a^	1.47 ± 0.07^a^	1.32 ± 0.12^a^
*Occludin*	1.00 ± 0.06	1.26 ± 0.20	1.49 ± 0.23	1.29 ± 0.12
*ZO-*1	1.00 ± 0.02	0.98 ± 0.07	1.17 ± 0.14	1.18 ± 0.14
**Day 42**				
IL-1β	1.00 ± 0.15^b^	3.43 ± 0.34^a^	3.63 ± 0.33^a^	4.12 ± 0.35^a^
IL-2	1.00 ± 0.18^b^	2.74 ± 0.26^a^	3.50 ± 0.75^a^	2.83 ± 0.46^a^
IL-6	1.00 ± 0.14^b^	1.09 ± 0.11^a^^b^	1.42 ± 0.10^a^	1.24 ± 0.12^a^^b^
IL-10	1.00 ± 0.05^c^	2.22 ± 0.15^b^	5.07 ± 0.59^a^	4.08 ± 0.50^a^
IFN-α	1.00 ± 0.19	1.20 ± 0.30	1.24 ± 0.09	1.64 ± 0.23
TNF-α	1.00 ± 0.05^c^	2.18 ± 0.11^b^^c^	3.73 ± 0.45^a^	2.81 ± 0.51^b^
TLR-4	1.00 ± 0.14^b^	1.83 ± 0.26^a^	2.08 ± 0.15^a^	2.23 ± 0.32^a^
*E-cadherin*	1.00 ± 0.14^c^	1.80 ± 0.03^a^^b^	1.55 ± 0.09^b^	1.93 ± 0.07^a^
*Occludin*	1.00 ± 0.12^b^	1.36 ± 0.13^a^^b^	1.34 ± 0.15^a^^b^	1.79 ± 0.22^a^
*ZO-*1	1.00 ± 0.14^b^	1.35 ± 0.14^a^^b^	1.51 ± 0.12^a^	1.41 ± 0.03^a^

*Data are presented as means ± SE, n = 5*.

a,b,c *Mean values within the same row with different superscript letters were significantly different (P < 0.05). IL, interleukin; IFN-α, Interferon-alpha; TLR, toll-like receptor; TNF-α, tumor necrosis factor-alpha; ZO-1, zonula occludens-1. Control group, a basal diet; BS-A group, a basal diet supplemented with B. subtilis A at 1 × 10^6^ CFU/g feed; BS-B group, a basal diet supplemented with B. subtilis B at 1 × 10^6^ CFU/g feed; and BS-C group, a basal diet supplemented with B. subtilis C at 1 × 10^6^ CFU/g feed*.

## Discussion

Our data clearly show that dietary supplementation with *B. subtilis* type A, type B, or type C increased the body weight and ADG of piglets in conjunction with a decrease in the diarrhea rate when compared with the control group. Weaning stress may give rise to imbalanced intestinal micro-ecology, high diarrhea incidence, and subsequently a low growth rate (27). These problems can be mitigated by dietary probiotics supplementation in pigs as demonstrated in several previous works (28). Our study confirms previous studies reporting positive effects of dietary supplementation with *B. subtilis* on growth performance in weanling piglets (8, 29, 30) and gives several important information regarding the biological events by which supplementating *B. subtilis* may exert beneficial effects. However, literatures on the effect of *B. subtilis* supplementation on the host is not fully homogeneous (11), likely because of different experimental designs.

Improved growth performance may be related, at least in part, to a reduced diarrhea rate, as observed in the present study. In good accordance with our results, previous studies also demonstrated that dietary supplementation with *B. subtilis* KN-42 reduced the incidence of diarrhea in weaned piglets (7), and *B. subtilis* WS-1 also reduced diarrhea rate and death rate caused by pathogenic *Escherichia coli* (31). It is known that weanling stress is associated with an increase of the CD and with a reduction in VH (32). Our data showed that dietary *B. subtilis* supplementation increased the VH and the VH/CD in the jejunum and ileum, suggesting that such supplementation would counteract the negative effects of the weanling transition on the structure of the intestinal epithelium.

The possible causal link between improved intestinal epithelium structure and decreased diarrhea rate and associated improved piglet's growth, that has been already proposed (9), is reinforced by the results of our study. Improved intestinal epithelium structure is associated with the increased ability of the small intestine to absorb nutrients in the luminal content (33). Such a beneficial effect of the supplementation with *B. subtilis* was accompanied by the up-regulated expression of E-*cadherin, occludin*, and *ZO*-1 in the colon. The E-*cadherin, occludin*, and *ZO*-1 are proteins involved in tight junction assembly, stability, and thus optimal for barrier function (34). Up-regulation of the expression of genes coding for *occludin* and *ZO*-1 were also observed *in vitro* using a porcine epithelial cell line in response to *B. subtilis* treatment (35).

Weaning is usually associated with substantial dynamic changes of the intestinal microbiota that may affect intestinal functions (36). Probiotics treatment may induce beneficial effects by acting on the intestinal ecosystem (37). In the present study, although Simpson or Shannon indexes did not differ throughout the trial, the relative abundances of *Anaerovibrio* and *Bulleidia* were increased while *Coprococcus* and *Clostridium* abundances were decreased in response to dietary *B. subtilis* supplementation. *Bulleidia* can degrade carbohydrates and promote the digestion and utilization of solid feeds (38), and reduced numbers of *Bulleidia* may accelerate the onset of diarrhea in weaned piglets (39). *Clostridium* is closely related to protein fermentation and can increase the risk of diarrhea (40). Therefore, modification of the microbiota ecosystem (increased *Bulleidia* and decreased *Clostridium*) might be another reason for the better growth performance and lower diarrhea rate in response to dietary *B. subtilis* supplementation as observed in the present study.

The SCFAs produced by colonic microbial fermentation of indigestible fiber are important for gut integrity, glucose homeostasis, and immune function (41). In the present study, acetate and propionate were the major SCFAs produced in the colon after the addition of *B. subtilis* in piglets' diets, which was consistent with previous findings in pregnant Huanjiang mini-pigs (22). Acetate can inhibit pathogenic bacteria, and butyrate acts as a major energy source for colonic epithelial cells (41), and as an important regulator of gene expression in colonocytes (42). BCFAs are produced by microbes through the deamination and decarboxylation of amino acids (43). Our results showed that dietary *B. subtilis* supplementation decreased the concentrations of isobutyrate and isovalerate. Since BCFA concentrations are considered as an indicator of protein catabolism (44), our findings strongly suggest that *B. subtilis* supplementation decreases protein fermentation in the intestinal luminal content. Since protein fermentation has been linked with increased production of amino acid-derived bacterial metabolites with adverse effects on the colonic epithelium (45), the beneficial effect of *B. subtilis* supplementation may be linked to its capacity to limit such protein fermentation by the intestinal microbiota.

Cytokines play crucial roles in the regulation of the immune and inflammatory responses and the barrier integrity of the gut (46). IL-2 is critical for regulating lymphoid homeostasis (47). IL-10, a regulatory anti-inflammatory cytokine, can prevent over-activation of the immune response and suppress the production of pro-inflammatory cytokines (48). TNF-α, a pro-inflammatory cytokine, can increase intestinal permeability through the dysregulation of tight junction proteins (49). In the present study, dietary *B. subtilis* supplementation increased the expression levels of IL-2 and IL-10 and decreased the expression level of TNF-α on day 21 of the trial. We thus propose that the decreased pro-inflammatory and increased anti-inflammatory cytokines caused by *B. subtilis* treatment may be linked to the capacity of these bacteria to decrease the increased inflammatory status associated with the weanling transition. A previous study also showed that probiotic *Lactobacillus* and *Bifidobacterium* can decrease plasma TNF-α level and increase IL-10 level (50). Moreover, the present study also found that dietatry BS-B supplementation increased TNF-α level on day 7 of the trial and dietary BS-C supplementation increased IL-6 level on day 21 of the trial, these effects being likely detrimental in reference with the pro-inflammatory nature of these cytokines. However, further studies are needed to reveal the underlying mechanisms that are responsible for these observations.

## Conclusion

In summary, dietary supplementation with BS-B or BS-C can improve the growth performance of piglets. This effect is associated with and maybe explained, by beneficial effects of *B. subtilis* on the diarrhea incidence, on the intestinal epithelium structure, and on the microbiota composition and metabolic activity in a sense that is believed to be associated with positive effects on gut health. We believe that these findings obtained are likely to be of interest and practical application for pig production.

## Data Availability Statement

The raw Illumina pair-end read data for all samples have been deposited in NCBI Sequence Read Archive (SRA) database under accession number: PRJNA597575.

## Ethics Statement

The experiments in the current study were approved by the Animal Welfare Committee of the Institute of Subtropical Agriculture, Chinese Academy of Sciences. The ethic approval number is ISA-2017-031.

## Author Contributions

XW, YZ, and WZ performed the experiments. ZT, XW, YD, MA, and FB performed the statistical analyses and wrote the manuscript. XK and ZW contributed to experimental concepts and design, provided scientific direction, and finalized the manuscript. All authors read and approved the final manuscript.

## Conflict of Interest

WZ was employed by the company Evonik (China). The remaining authors declare that the present study was conducted in the absence of any commercial or financial relationships that could be construed as a potential conflict of interest.
