# Supplementary material for: Dietary Supplementation With Bacillus subtilis Promotes Growth and Gut Health of Weaned Piglets
Source: Front Vet Sci. 2021 Jan 15;7:600772. doi: 10.3389/fvets.2020.600772 (PMC7844206; doi:10.3389/fvets.2020.600772)
Supplement: Supplementary file 1 [file Table_1.pdf]

Supplementary Material

Supplementary Table 1 Composition and nutrient levels of basal diets of piglets (as-fed basis)

Items	Early nursery diet (28-48 days of age)	Late nursery diet (49-69 days of age)
Ingredients		
Corn	22.00	69.50
Broken rice	25.00	-
Wheat flour	12.00	-
Glucose	3.00	-
Soybean meal (46% CP)	10.50	-
Soybean meal (43% CP)	-	16.00
Puffed soybean	10.00	-
Fermented soybean	2.50	4.00
Soybean protein concentrate	-	2.00
Fish meal	3.00	1.00
Low-protein whey power	5.00	-
Egg power	0.50	-
Wheat bran	-	2.00
Soybean oil	1.00	1.50
Citric acid	1.50	-
Early nursery Premix [1)]	4.00	-
Late nursery Premix [2)]	-	4.00
Total	100.00	100.00
Nutrient level [3)]	-	-
Energy (MJ/Kg)	14.24	13.83
Crude protein	24.50	20.65
Crude fat	3.10	2.33
Calcium	0.81	0.97
Total phosphorus	0.53	0.55
Lysine	1.36	1.18
Methionine	0.50	0.39
Threonine	0.87	0.72

[1),2)] The premix compositions were in accordance with NRC (2012) recommended nutrient requirements for growing and fattening pigs. [3)] Energy is a calculated value while the others are measured values.
