# Supplementary material for: Dietary Supplementation With Bacillus subtilis Promotes Growth and Gut Health of Weaned Piglets
Source: Front Vet Sci. 2021 Jan 15;7:600772. doi: 10.3389/fvets.2020.600772 (PMC7844206; doi:10.3389/fvets.2020.600772)
Supplement: Supplementary file 2 [file Table_2.pdf]

Supplementary Material

Supplementary Table 2 Primers for real-time PCR analysis

Gene name	Sequence (5'-3')		Product size (bp)
E-cadherin	F:GAAGGAGGTGGAGAAGAGGAC	R:AGAGTCATAAGGTGGGGCAGT	216
IL-1β	F: GCTAACTACGGTGACAACAA	R: TCTTCATCGGCTTCTCCACT	196
IL-2	F: TGCACTAACCCTTGCACTCA	R: CAACTGTAAATCCAGCAGCAA	100
IL-6	F: TCCAGCATCATTGCATCATC	R: GGCTCCACTCACTCCACAAG	105
IL-10	F:GGGCTATTTGTCCTGACTGC	R:GGGCTCCCTAGTTTCTCTTCC	105
IFN-α	F:CCTGGACCACAGAAGGGA	R:TCTCATGCACCAGAGCCA	92
Occludin	F:ATGCCTCCTCCCCTTTCG	R:CGCCCGTCGTGTAGTCTGTC	295
TLR-4	F:CAGATAAGCGAGGCCGTCATT	R:TTGCAGCCCACAAAAAGCA	113
TNFα	F: ACAGGCCAGCTCCCTCTTAT	R: CCTCGCCCTCCTGAATAAAT	102
ZO-1	F:TACCCTGCGGCTGGAAGA	R:GGACGGGACCTGCTCATAACT	154
β-actin	F: CTGCGGCATCCACGAAACT	R: AGGGCCGTGATCTCCTTCTG	147
