# Supplementary material for: Dietary Supplementation With Bacillus subtilis Promotes Growth and Gut Health of Weaned Piglets
Source: Front Vet Sci. 2021 Jan 15;7:600772. doi: 10.3389/fvets.2020.600772 (PMC7844206; doi:10.3389/fvets.2020.600772)
Supplement: Supplementary file 3 [file Table_3.pdf]

Supplementary Material

Supplementary Table 3 Effect of dietary supplementation with different types of *B. subtilis* on gut metabolites of weaned piglets

Items	Control group	BS-A group	BS-B group	BS-C group
Day 7				
Tryptamine	0.09±0.06	0.04±0.02	0.03±0.01	0.06±0.02
Phenylethylamine	0.28±0.10	0.31±0.12	0.50±0.22	0.20±0.08
Putrescine	0.81±0.10	0.67±0.37	0.78±0.48	0.23±0.03
Tyramine	1.15±0.53	0.92±0.50	0.34±0.10	0.65±0.17
Spermidine	2.98±0.38	3.23±0.64	1.88±0.51	2.40±0.44
Spermine	6.16±2.03	4.53±1.92	4.78±2.04	1.85±0.62
Indole	8.21±1.29	6.48±0.99	5.12±1.00	4.90±0.75
Skatole	3.99±0.98	2.38±0.29	4.51±1.33	3.14±0.75
Day 21				
Tryptamine	$0.13±0.03^{b}$	$0.05±0.02^{ab}$	$0.06±0.04^{ab}$	$0.01±0.01^{a}$
Phenylethylamine	0.07±0.05	0.22±0.13	0.20±0.10	0.13±0.05
Putrescine	3.91±1.85	1.90±0.93	1.91±1.19	1.02±0.55
Tyramine	$0.37±0.09^{ab}$	$0.77±0.27^{b}$	$0.36±0.17^{ab}$	$0.15±0.08^{a}$
Spermidine	3.78±1.06	3.27±0.74	2.58±0.67	3.71±0.44
Spermine	2.44±1.48	2.07±0.57	3.38±1.06	3.80±1.31
Indole	2.66±0.48	3.43±1.37	2.05±0.47	3.21±0.68
Skatole	$6.82±0.75^{b}$	$2.89±0.40^{a}$	$5.12±1.13^{ab}$	$4.63±1.28^{ab}$
Day 42				
Tryptamine	1.38±0.34	1.54±0.11	1.26±0.31	1.61±0.24
Phenylethylamine	0.00±0.00	0.08±0.04	0.11±0.05	0.18±0.09
Putrescine	15.44±1.60	15.38±3.15	13.44±2.22	12.99±1.95
Tyramine	3.09±0.54	1.94±0.60	3.00±0.96	3.64±0.16
Spermidine	21.23±1.38	18.97±2.82	19.38±2.89	18.33±2.70
Spermine	4.01±0.50	2.99±0.38	3.98±1.06	4.55±0.79
Indole	$3.70±0.90^{a}$	$7.32±0.70^{b}$	$5.73±1.21^{ab}$	$4.54±1.25^{ab}$
Skatole	$11.59±3.50^{b}$	$5.07±1.41^{a}$	$4.32±0.62^{a}$	$4.26±1.07^{a}$

Data are expressed as means ± SE, $n = 5$. ^{a,b} Mean values within the same row with different superscript letters were significantly different ($P < 0.05$).
